# Weight Loss of Over 100 lbs in a Patient of Prader-Willi Syndrome Treated With Glucagon-Like Peptide-1 (GLP-1) Agonists

**DOI:** 10.7759/cureus.35102

**Published:** 2023-02-17

**Authors:** Sana Ahmed, Arooj Naz, Mahnoor K

**Affiliations:** 1 Internal Medicine, Smt. Kashibai Navale Medical College, Mumbai, IND; 2 Medicine, Frontier Medical and Dental College, Abbottabad, PAK; 3 Internal Medicine, Fauji Foundation University, Islamabad, PAK; 4 Internal Medicine, Foundation University Medical College, Islamabad, PAK

**Keywords:** exenatide, liraglutide or saxenda, glp-1 receptor agonist, weight loss and obesity, prader-willi syndrome

## Abstract

Prader-Willi syndrome (PWS) is the most common genetic obesity syndrome. The clinical features of this condition include childhood obesity, hyperphagia, infantile hypotonia, hypogonadism, short stature, and characteristic facial features. The leading cause of morbidity and mortality in PWS is hyperphagia and resultant obesity. Here, we highlight the effectiveness of glucagon-like peptide-1 (GLP-1) agonists by reporting an interesting case of successful rapid weight loss in an adult with PWS using GLP-1 agonists - exenatide and liraglutide. To the best of our knowledge, this report presents the first clinical evidence supporting the use of GLP-1 receptor agonists in the treatment of genetic obesity syndromes; our patient lost a total of 125 lbs on GLP-1 analog and continues to lose weight.

## Introduction

There are several genetic obesity syndromes; Prader-Willi syndrome (PWS) is the most common among them, with an incidence of 1:10,000 to 1:30,000 at birth [1]. PWS is a neurodevelopmental genomic imprinting disorder characterized by a lack of gene expression from paternal chromosome 15 [2]. The clinical features of this condition include childhood obesity, hyperphagia, infantile hypotonia, hypogonadism, short stature, and characteristic facial features [3]. Its diagnosis is confirmed by genetic testing, and the treatment mainly involves supportive measures. The leading cause of morbidity and mortality in PWS is hyperphagia and resultant obesity. Obesity is the primary target of available treatment regimens [4]. Apart from nutritional and behavioral management, several pharmacological options have been studied. Here, we highlight the effectiveness of glucagon-like peptide-1 (GLP-1) agonists by reporting a compelling case of successful rapid weight loss in an adult using GLP-1 agonists - exenatide and liraglutide. GLP-1 receptors function by increasing insulin secretion and inhibiting glucagon post-meals. They also inhibit gastric emptying, increasing nutrient absorption and decreasing food intake, thereby limiting weight gain [5]. Unlike endogenous GLP-1 molecules, liraglutide has a fatty acid molecule resulting in a prolonged half-life and, subsequently, a lengthier effect. The medication is commonly administered subcutaneously with a half-life of 13 hours and is administered once daily. Its maximum concentration is reached 8-12 hours after administration [6].

## Case presentation

A 27-year-old Hispanic male with a history of PWS, obesity, diabetes mellitus type II, and sleep apnea presented to the endocrinology outpatient clinic in September 2012 for diabetes control and obesity. He weighed 350 lbs (158.75 kg) with a body mass index (BMI) of 106.8. He had no family history of type 2 diabetes mellitus. His temperature was 36.6 °C, and his blood pressure was 132/76 mmHg. He had a pulse of 70/minute, and a respiratory rate of 20/minute with an O_2 _saturation of 93% on room air. Physical examinations revealed difficulty ambulating and hyperpigmentation over the neck and axillary regions. His hemoglobin A1C (HbA1c) was 7.4%., and his fasting glucose level was 172 mg/dl. Laboratory tests including free T4, human growth hormone, cortisol, 24-hour urine-free cortisol, adrenocorticotropic hormone (ACTH), follicle-stimulating hormone (FSH), luteinizing hormone (LH), prolactin, estradiol, progesterone, thyroid, testosterone, and dehydroepiandrosterone sulfate (DHEA-S) were within normal limits.

**Figure 1:**
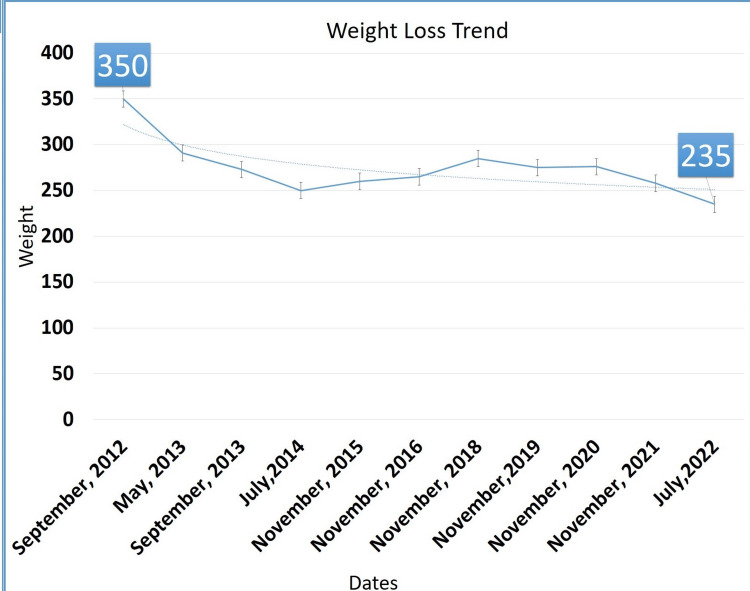
Weight-loss trend

Under the diagnosis of morbid obesity and diabetes mellitus, a dietitian was consulted, who recommended a high-fiber diabetic diet with a calorie restriction of 1800 kcal/day. In addition to diet control with lifestyle modifications, pharmacological treatment with GLP-1 receptor agonist (exenatide 2 mg/day via subcutaneous injection) was initiated. Following the initiation of exenatide, the patient experienced weight loss, which continued throughout the therapy until October 2014. Twenty-four months of treatment led to the reduction of his HbA1c level to 5.7% from an initial level of 7.4%, while his weight dropped from 350 lbs to 250 lbs, as seen in Figure 1. There was a total weight loss of around 100 lbs and a 2% drop in HbA1c. However, he reported poor compliance with the twice-daily dosage of exenatide. Therefore exenatide was replaced with liraglutide (1.2 mg/day via subcutaneous injection). He reported no gastrointestinal distress or hypoglycemia problems and noted a slight decrease in appetite. He is currently under regular outpatient clinic follow-up. The weight in July 2022 was 235 lbs, revealing 125 lbs of weight loss since September 2012, associated with the use of GLP-1 analog and a restrictive 1800-calorie diet, as seen in Table 1.

**Table 1:** Weight changes attributed to using glucagon-like peptide-1 (GLP-1) agonists

Time frame	Recorded weight (lbs)	Weight-loss trajectory (lbs)
September 2012	350	
May 2013	291	-59
September 2013	273	-18
July 2014	250	-23
November 2015	260	-10
November 2016	265	-5
November 2018	285	+20
November 2019	275	+10
November 2020	276	+1
November 2021	258	-18
July 2022	235	-23
Total weight loss to date		125 lbs

## Discussion

As noted in this case report, GLP-1 receptor agonists significantly reduced HbA1c levels and led to weight loss in a pathologically overweight individual with significantly elevated blood sugars. This case illustrates significant continuous weight loss, amounting to a total of 125 lbs (52.163 kg). GLP reduces appetite in individuals with both normal BMI and obesity (BMI >25) [6]. The primary mechanism of action is delayed gastric emptying and increased adipocyte metabolism.

Appetite and satiety are regulated by an intricate relationship between gastric and hypothalamic hormones. Gastric activity is affected by two incretin mimetics; glucose-dependent insulinotropic peptide (GIP) and GLP-1. Immediately after food intake, enteroendocrine K cells in the gut, especially in the duodenum and jejunum, secrete GIP. GIP binds to its receptor leading to an intracellular increase in cAMP and Ca2+ levels from β cells, resulting in adipocyte metabolism. GLP-1, secreted by enteroendocrine L cells primarily in the small bowel and ascending colon, has a similar effect on cAMP and Ca2+ levels but works to inhibit gastric emptying. This contributes to decreased food intake due to increased satiety. GLP-1 also stimulates pancreatic β-cell proliferation by upregulating homeobox-1 protein, which leads to increased insulin gene transcription and GLUT2 receptor upregulation in the liver, pancreatic, intestinal, kidney cells, and hypothalamus [7]. The hypothalamus is responsible for secreting two hormones, among many others, that play a crucial role in weight changes: leptin and ghrelin. Leptin primarily regulates energy and increases metabolism [8], leading to a reduction in weight. In contrast, ghrelin is an appetite stimulator and leads to increased food intake [9]. The overall effect of GIP and GLP-1 leads to an increase in leptin and a decrease in ghrelin secretion, which results in decreased weight.

Specifically, in our case of a patient with PWS, the weight loss after using liraglutide was substantial. Apart from weight loss, the patient also showed clinical improvement with ambulation and experienced a decrease in HbA1c to 5.7%, reducing his risk of diabetes and its associated complications. From 2018 to 2020, the patient was enrolled in a healthcare facility where his diet was not tailored to his specific needs, accounting for the increase in the weight he gained during that period.

## Conclusions

In genetically obese individuals, healthy body weight is considered the primary goal of treatment, but managing weight loss is often a challenging hurdle; despite consuming the same amount of calories as any other person; sarcopenia and decreased resting energy expenditure (REE) cause them to gain weight. Therefore, a thoroughly planned diet and nutritional management are of utmost importance in these cases. In obesity syndrome patients, pharmacotherapy options are limited. Pharmacological options include drugs targeting lipid digestion and absorption, carbohydrate metabolism, or increased energy expenditure. Examples of such medications include orlistat, metformin, and sibutramine, respectively. Other medicines that increase energy expenditure include naltrexone and lorcaserin. Behavioral changes, such as those related to seeking food, can be accomplished using topiramate or somatostatin analogs. Patients with diabetes mellitus type II have experienced considerable benefits with GLP-1 agonists concerning weight, satiety, and glycemic control, especially in cases of PWS. Our patient comfortably tolerated the course for 10 years and lost nearly 125 lbs.

In conclusion, there are considerable benefits associated with GLP-1 agonists in treating obesity in genetic obesity syndromes. Early initiation of multidisciplinary care and parental education enables obesity syndrome patients to keep their weight within a healthy range, thereby improving their quality of life. In this patient, the GLP-1 agonist has shown promising results in long-term use, thus enabling further investigation into the benefits of the GLP-1 agonist for weight control in other genetic obesity syndromes. The authors hope this case provides insights into the benefit of using GLP-1 agonists for genetic obesity syndromes.
